# Emotional Eating and Its Relationship with Symptoms of Anxiety, Depression, and Stress During the COVID-19 Pandemic: A Multicenter Study in College Students

**DOI:** 10.3390/ijerph22030354

**Published:** 2025-02-27

**Authors:** Isabela Silva, Adriana Lúcia Meireles, Carolina Martins dos Santos Chagas, Clareci Silva Cardoso, Helian Nunes de Oliveira, Eulilian Dias de Freitas, Fernanda de Carvalho Vidigal, Luciana Neri Nobre, Luciana Saraiva da Silva, Waléria de Paula, Lívia Garcia Ferreira

**Affiliations:** 1 Graduate Program in Nutrition and Health, Department of Nutrition, Universidade Federal de Lavras (UFLA), Lavras 37200-900, MG, Brazil; isabela0silva@yahoo.com.br (I.S.); carolinachagas@ufla.br (C.M.d.S.C.); 2 Department of Clinical and Social Nutrition, School of Nutrition, Universidade Federal de Ouro Preto (UFOP), Ouro Preto 25400-000, MG, Brazil; adriana.meireles@ufop.edu.br; 3 Graduate Program in Health Sciences, Department of Public Health, Universidade Federal de São João Del-Rei (UFSJ), Divinópolis 35501-296, MG, Brazil; clarecicardoso@yahoo.com.br; 4 Medical School, Department of Social and Preventive Medicine, Universidade Federal de Minas Gerais (UFMG), Belo Horizonte 30130-100, MG, Brazil; heliannunes@gmail.com; 5 Department of Medicine, Universidade Federal de Juiz de Fora, Campus Avançado Governador Valadares (UFJF-GV), Governador Valadares 35020-220, MG, Brazil; eulilian.freitas@ufjf.br; 6 Faculty of Nutrition, Universidade Federal de Alfenas (UNIFAL), Alfenas 37130-001, MG, Brazil; fernanda.vidigal@unifal-mg.edu.br; 7 Graduate Program in Sciences of Nutrition, Department of Nutrition, Universidade Federal dos Vales do Jequitinhonha e Mucuri, Campus JK (UFVJM), Diamantina 39100-000, MG, Brazil; luciana.nobre@ufvjm.edu.br; 8 School of Medicine, Universidade Federal de Uberlândia (UFU), Uberlândia 38405-320, MG, Brazil; luciana.saraiva@ufu.br; 9 Graduate Program in Pharmaceutical Sciences, School of Pharmacy, Universidade Federal de Ouro Preto (UFOP), Ouro Preto 35400-000, MG, Brazil; waleria.paula@aluno.ufop.edu.br

**Keywords:** Brazil, college students, eating behavior, mental disorders

## Abstract

Emotional eating refers to overeating triggered by negative emotions, and it is common among those with mental health challenges. Academics are vulnerable to changes in eating habits and mental well-being, especially after the pandemic began. This study aimed to analyze the relationship between emotional eating and the signs and symptoms of mental disorders in undergraduates aged 18 and older. They completed a virtual questionnaire assessing emotional eating using the Three-Factor Eating Questionnaire, and mental health by a reduced version of the Depression Anxiety and Stress Scale. In this cross-sectional, multicenter survey, 8650 students (65.7% female, average age 22) participated, with a median body mass index of 23.3 kg/m^2^, and 23.8% were enrolled in health-related courses. The median emotional eating score was 44.4 (22.2–66.7), and a large proportion had symptoms of depression (73.2%), stress (68.1%), and anxiety (66.1%). Symptoms of anxiety (CI = 1.291–1.714), depression (CI = 1.469–1.983), and stress (CI = 1.483–2.006) were independently associated with higher emotional eating scores (≥66.7) in multiple regression analyses. Based on the significant associations found, the importance of integrating mental health support and intervention strategies, such as psychological counseling and nutritional guidance, to mitigate emotional eating among university students is highlighted, along with the need for future studies to explore the causal relationships.

## 1. Introduction

Emotional eating is considered a behavior of eating disinhibition, characterized by a tendency toward uncontrolled food consumption in response to emotional factors, both positive and negative [1,2]. This behavior is frequently linked to stress and anxiety, as it affects emotional regulation and influences food preferences [3,4]. When emotional eating becomes dysfunctional and is associated with overeating, it can contribute to unhealthy eating patterns, such as binge eating episodes, weight fluctuations, and an increased risk of developing eating disorders and mental health issues [5,6,7,8,9].

In this context, academic life emerges as a significant stressor that can further intensify these effects driven by the demands of academic routine, social adaptation, and future career concerns [10,11]. The combination of these stressors can increase the likelihood of students turning to food as a coping mechanism for emotional distress, which may lead to the consumption of comfort foods high in fat and sugar [3,4]. Additionally, university students are often exposed to environments that encourage unhealthy food choices, such as late-night study sessions, social gatherings, and limited access to balanced meals, all of which can exacerbate emotional eating tendencies [12].

The transition to university life also involves significant lifestyle changes, reduced physical activity, disruptions in sleep patterns, academic pressures, and potential financial constraints, factors that contribute to the development or worsening of mental health issues [10,11]. Symptoms such as anxiety (e.g., tachycardia, nausea, and insomnia), depression (e.g., low mood, social isolation, irritability, and persistent negative thoughts), and stress (e.g., feeling overwhelmed, and exposure to stressful situations) [13] are closely linked to the overall health and well-being of young individuals [14,15] as well as their academic performance [16].

Furthermore, the unprecedented impact of the COVID-19 pandemic intensified these challenges, introducing additional stressors such as prolonged social isolation and remote learning, which intensified the relationship between psychological distress and emotional eating, among university students [17].

These factors can lead to dysfunctional emotional eating, which has been identified as an important contributor to the development or exacerbation of eating disorders. In the course of early phases of the pandemic, weight gain, increased body dissatisfaction, and stress levels heightened the likelihood of Brazilian university students developing disordered eating behaviors [18]. Moreover, emotional eating during the pandemic was found to be associated with increased body dissatisfaction and stress among this population [19].

While the existing literature documents emotional eating and some symptoms of mental health disorders separately, a clear gap remains in understanding the specific mechanisms that link these patterns in Brazilian university students [20,21,22,23]. Prior studies have primarily examined these factors in a limited way, without fully exploring their interconnectedness, especially within the context of a global crisis such as the COVID-19 pandemic [14,24]. Additionally, many studies focus on specific subgroups, such as students from health-related fields [15,25] or particular stages of the academic curriculum [14]. They often rely on small sample sizes or restrict the analysis to a single university or city, limiting the generalizability of findings to the broader university population [26,27,28,29].

This study aims to address this gap by investigating the association between emotional eating and symptoms of anxiety, depression, and stress in a large sample of Brazilian university students during the COVID-19 pandemic. By encompassing multiple institutions and various academic courses, this research provides a more comprehensive perspective on how psychological issues influence eating behaviors in young adults. Understanding these associations is critical for developing targeted interventions that address both mental health and eating behaviors within university settings.

The hypothesis of this study is that symptoms of mental health disorder are related to emotional eating. Additionally, students who exhibit higher levels of anxiety, depression, and stress are expected to display greater tendencies toward emotional eating. By providing evidence of this relationship, the study may contribute to the development or adaptation of mental health and nutritional policies in higher education institutions, particularly in the aftermath of the COVID-19 pandemic.

## 2. Materials and Methods

### 2.1. Study Design

The multicenter epidemiological survey, titled Project on Anxiety and Depression in University Students (PADu), is a cross-sectional study conducted via an online survey due to the COVID-19 pandemic. This study involved the participation of eight Federal Institutions of Higher Education located in Minas Gerais: Federal University of Lavras (UFLA), Federal University of Juiz de Fora (UFJF), Federal University of Minas Gerais (UFMG), Federal University of Ouro Preto (UFOP), Federal University of São João Del-Rei (UFSJ), Federal University of Uberlândia (UFU), Federal University of Alfenas (UNIFAL), and Federal University of Vales do Jequitinhonha and Mucuri (UFVJM). The questionnaire was accessible from October 2021 to February 2022, and the data collection period at each university spanned three months.

The research team received prior training on the virtual questionnaire used. The primary focus of PADu is academics’ mental health, and this specific study examines the relationship between symptoms of mental health and emotional eating. Responsibilities for the dissemination of the research, data collection, and project monitoring were allocated to Master’s, Doctoral, and Scientific Initiation students. The survey was disseminated through the students’ institutional e-mail, during remote classes, and on social media, especially the Instagram^®^ platform.

### 2.2. Sample

For sample selection, this study started with a total of 118,828 university students, representing the sum of all students enrolled in the institutions involved. Considering the unknown actual frequency of the outcome (in this case, emotional eating), a hypothetical frequency of 50% was employed, as suggested by the OpenEpi^®^ program. Additionally, a confidence limit of 2% and a design effect of 1.0, indicating a random sample, albeit for convenience, were employed. To fulfill these criteria with a confidence interval of 99.9%, the required sample size amounted to 6403 students.

The sample selection was performed by a nonprobabilistic method, and the inclusion criteria were male and female volunteer students enrolled in face-to-face undergraduate courses at universities, aged 18 years or older. Excluded from the study were individuals who did not complete the virtual questionnaire and students regularly enrolled in the undergraduate exchange program.

The survey link was sent to 118,828 eligible students and, considering the nature of the sampling method, a response rate of approximately 7.3% was achieved.

### 2.3. Procedures

Participants answered the virtual questionnaire on the Google Forms^®^ platform in approximately 30 min. The variables of interest in this study were emotional eating, signs and symptoms of mental disorders, personal data (such as sex, age, and monthly family income), physical activity, ultra-processed food (UPF) consumption, estimated body mass index (BMI), and course area.

Family income was reported in brackets corresponding to multiples of the minimum wage. The median of each bracket was extracted and used to represent the family income. Physical activity was assessed based on the three months preceding the study and could include activities such as running, cycling, walking, weightlifting, and yoga, among others. The UFP consumption was analyzed over the past 30 days using a food frequency questionnaire which included the intake of packaged snacks, instant noodles, processed meats, frozen products, canned goods, bread, and sweets [30]. The frequency of physical activity and UFP consumption was classified as ‘no’ if the reported frequency was never or almost never; and ‘yes’ if the ranged from once per week to every day.

BMI calculations relied on self-reported weight and height provided by the students. Classification followed the World Health Organization (WHO) recommendations for adolescents aged up to 20 years [31] and adults [32], and Lipschitz (1994) [33] for elderly individuals. Courses of study were categorized based on knowledge areas [34] and classified into health and other areas.

### 2.4. Instruments

#### 2.4.1. Emotional Eating

The original Three-Factor Eating Questionnaire has 51 items, but the short version (TFEQ-21) was used to analyze the eating behavior profile according to the emotional eating domain score. The TFEQ-21 subscale was selected to assess eating behavior because it is the only instrument translated and validated for the Brazilian population. The translated and validated version designed for the Brazilian population was used and had adequate internal consistency with Cronbach’s alpha of 0.85 [1]. For this investigation, only the six items (questions 2, 4, 7, 10, 14, and 16) of the questionnaire related to the independent TFEQ domain of emotional eating were applied, as indicated by the scale authors.

Each item was answered on a scale ranging from 1 (“Definitely true”) to 4 (“Definitely false”). The score was obtained by reverse-coding the responses, calculating the mean, and transforming them into a numerical scale ranging from 0 to 100 points through a specific mathematical formula: {[(mean × 6) − 6]/18} × 100. A higher score on this subscale indicated a stronger inclination toward overeating in response to negative emotional stimuli [1]. Moreover, the emotional eating scale was divided into quartiles, with values above the third quartile (Q3 ≥ 66.7) as a cutoff point according to other authors [29,35]. The highest quartile was chosen because it represents the students with higher scores in emotional eating.

#### 2.4.2. Symptoms of Anxiety, Depression, and Stress

The assessment of mental disorders’ symptoms utilized the reduced version of the Depression Anxiety and Stress Scale (DASS-21), translated and validated for the Brazilian population with a Cronbach’s alpha of 0.92 [36]. The scale’s purpose was to screen for signs and symptoms and did not provide a diagnosis. Comprising 21 questions, this version evaluated each disorder—anxiety, depression, and stress—via seven questions that inquired about symptoms experienced over the preceding week [36,37].

Each item was assessed on a scale ranging from 0 (“Did not apply to me at all”) to 3 (“Applied to me very much, or most of the time”). To determine severity levels, the scores corresponding to symptoms of anxiety, depression, and stress were summed and multiplied by two. The classification criteria were as follows: normal (0–7, 0–9, and 0–14, respectively), mild (8–9, 10–13, and 15–18, respectively), moderate (10–14, 14–20, and 19–25, respectively), severe (15–19, 21–27, and 26–33, respectively), and extremely severe (≥20, ≥28, and ≥34, respectively) [36]. This classification followed the questionnaire authors’ guidelines [36,37] and has also been observed in other studies [38,39].

In addition to the signs and symptoms of mental disorders, the individual was also asked about any previous diagnosis of anxiety and/or depression.

### 2.5. Statistical Analysis

The data were organized in an Excel spreadsheet and analyzed using the Statistical Package for Social Sciences (SPSS) version 25, with a significance level set at 0.05.

Categorical variables are expressed as absolute frequencies and percentages, while numerical variables are expressed as medians and interquartile ranges due to the non-normal distribution observed in the sample (Kolmogorov-Smirnov test). The internal consistency of the Emotional Eating-TFEQ and DASS-21 questionnaires used in this study was assessed using Cronbach’s alpha, yielding results of 0.92 and 0.86, respectively. These findings confirm that the instruments used in this study were appropriate for assessing emotional eating and symptoms of mental disorders in the sample.

To evaluate potential distinctions among emotional eating scores and categorical variables, including DASS-21 classification and personal data, Mann-Whitney and Kruskal-Wallis tests (with Dunn’s post hoc test) were utilized. The Spearman correlation was employed to assess the relationship between numerical variables—such as age, family income, and BMI value—and the emotional eating scores. Additionally, Chi-square tests were conducted for categorical variables, specifically between the classification of mental disorders and emotional eating ≥ 66.7 (Q3), with the observation of adjusted residuals obtained through post hoc pairwise analysis with Bonferroni correction [40].

Multiple logistic regression was performed using the insert method to analyze the factors independently associated with the higher scores of emotional eating [above the third quartile (Q3 ≥ 66.7)]. The variables sex, age, family income, BMI, course area, physical activity, and UPF consumption were used as adjustment variables and the model’s quality was assessed using the Hosmer-Lemeshow test (*p* > 0.05). Missing data for sex and BMI were excluded due to their low percentages (0.4% and 0.7%, respectively), while missing data for family income (6.5%) were handled using multiple imputation.

### 2.6. Experimental Methods

The PADu project was submitted to and approved by the Human Research Ethics Committee (HREC) of all participating universities. This study was conducted according to the guidelines laid down in the Declaration of Helsinki and all procedures involving human subjects were approved by the UFLA Human Research Ethics Committee (HREC) with protocol number 43027421.3.2006.5148. Written informed consent was obtained from all subjects.

## 3. Results

The surveyed sample comprised 8997 students from eight universities. However, 347 individuals did not meet the selection criteria because they were graduate students, did not complete the questionnaire, or were participating in an exchange program. Therefore, the valid sample consisted of 8650 students: 2002 from UFMG, 1806 from UFOP, 1530 from UFU, 909 from UFJF, 847 from UFVJM, 633 from UFLA, 476 from UFSJ, and 447 from UNIFAL.

Among the respondents, the majority were women (65.7%; *n* = 5660), the median age was 22.0 years (20.0–25.0), and 23.8% (*n* = 2061) of the participants were enrolled in health courses.

The median emotional eating score among all students was 44.4 (22.2–66.7) points. Women had higher median emotional eating than men (*p* < 0.01), and students enrolled in health area courses showed a significant association with emotional eating (*p* < 0.05). There was a positive correlation between emotional eating and both age and BMI (*p* < 0.01) (Table 1).

Most students showed signs and symptoms of depression (73.2%; *n* = 6330), stress (68.1%; *n* = 5889), and anxiety (66.1%; *n* = 5717). Among those with some symptomatic level, extremely severe anxiety and depression, as well as severe stress, were frequent, as shown in Table 2. Students who presented the integrated presence of all three symptoms of mental disorders (53.7%; *n* = 4643) exhibited higher scores of emotional eating [50.0 (33.3–77.8); *p* < 0.01] compared to those who had no symptoms [46.3%; *n* = 4007; 33.3 (16.7–55.6)].

**Figure 1:**
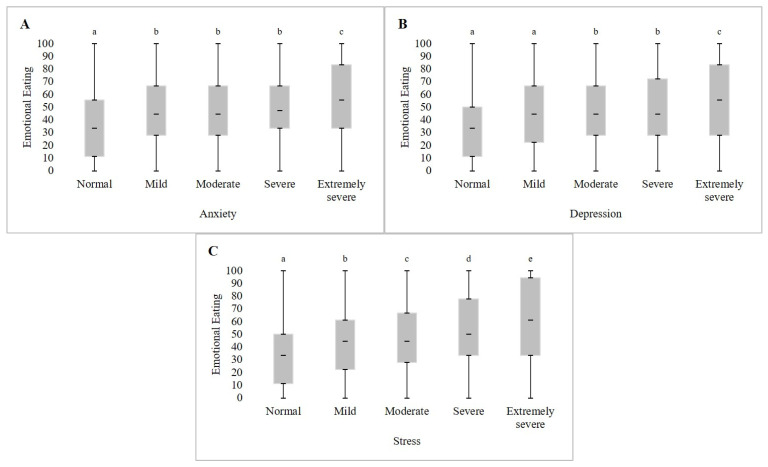
Median emotional eating scores and the classifications of signs and symptoms of anxiety (**A**), depression (**B**), and stress (**C**) of students enrolled in face-to-face undergraduate classes at Federal Institutions of Higher Education of Minas Gerais in 2021 and 2022. Legend: Different lowercase letters represent significantly different values through Kruskal-Wallis with Dunn’s post hoc test (*p* < 0.01).

Furthermore, in students with high scores in emotional eating (≥Q3), there is a statistically significant prevalence of students with signs and symptoms of extremely severe mental disorders (Table 2); and the individuals with extremely severe symptoms of mental disorders had higher scores on emotional eating than the others (*p* < 0.01) (Figure 1). The median emotional eating values for each level of signs and symptoms of mental disorders are in Table A1 (Appendix A).

Considering only students without a prior diagnosis of anxiety and depression (*n* = 3539), the aforementioned association remained significant; i.e., individuals with extremely severe symptoms of anxiety, depression, and stress had higher emotional eating scores compared to others (*p* < 0.01). Among all responding students (*n* = 8650), 28.7% (*n* = 2486) reported having received a previous diagnosis of both anxiety and depression. A diagnosis of anxiety was reported by 56.7% (*n* = 4906) of students, while depression was reported by 31.1% (*n* = 2691), though not exclusively. Moreover, upon analyzing individuals with a previous diagnosis of anxiety and/or depression (59%; *n* = 5111), those diagnosed with anxiety [50.0 (27.8–72.2)] or depression [50.0 (27.8–77.8)] showed significantly higher emotional eating scores compared to those without a diagnosis of anxiety [38.9 (16.7–61.1)] or depression [38.9 (22.2–66.7)], (*p* < 0.01).

In multiple regression analyses, having some symptoms (mild, moderate, severe, and extremely severe) of anxiety, depression, and stress were identified as independent factors associated with an increased likelihood of falling into the highest quartile of the emotional eating score. The data are summarized in Table 3.

## 4. Discussion

This multicenter epidemiological study found a significant association between emotional eating and signs and symptoms of anxiety, depression, and stress in university students during the pandemic. Furthermore, it demonstrated that, as these symptoms intensify, there is a corresponding increase in emotional eating scores. Eating behavior changes according to emotional state, and seeking food to relieve certain emotions is considered normal [4]. However, it can transition to dysfunctional or even pathological patterns, depending on the frequency, quantity, and quality of food consumed, the associated emotions, and the lingering emotional effects post-consumption [1].

The emotional eating domain of the TFEQ utilized in this study assesses the tendency to overeat when experiencing negative emotions. The rising score indicates progressive intensity in the emotional trigger for uncontrolled eating, whose high frequency can characterize compensatory and dysfunctional behavior (in this study, ≥Q3) [1]. In the present study, the presence of signs and symptoms of mental disorders were independent predictors of dysfunctional emotional eating.

The median score for emotional eating in the current study [44.4 (22.2–66.7)], performed during the pandemic of COVID-19, was similar to that of other researchers who also used the short version of TFEQ to evaluate emotional eating. Liboredo et al. (2021) [29] investigated eating behavior and perceived stress in adults (*n* = 1368) during the pandemic [44.5 points (27.8–61.1)]. Similarly, Lira et al. (2020) [21] explored the tendency toward eating disorders among undergraduate health students (*n* = 192) before the pandemic (42.4 ± 30.7). However, it is pertinent to highlight that those students surveyed by PADu with signs and symptoms of extremely severe mental disorders had a median emotional eating score of 55.6 (33.3–83.3) for anxiety, 55.6 (27.8–83.3) for depression, and 61.1 (33.3–94.4) for stress.

In the present study, 73.2% of the students presented some level of severity of depression, 68.1% of stress, and 66.1% of anxiety, showing higher rates compared to a prior study involving 1074 students conducted in the pre-pandemic period, where the frequency of depression, stress, and anxiety was 18.4%, 34.5%, and 23.6%, respectively [39]. Kavvadas et al. (2022) [38] evaluated 2110 students in November 2020 and 2916 in November 2021 and found frequencies of depression of 60.0% and 65.4%, stress of 49.8% and 59.3%, and anxiety of 40.0% and 55.6%, respectively. Such results indicate that the prevalence of mental disorders is high and that the scenario of the COVID-19 pandemic has intensified the signs and symptoms of psychiatric problems.

The university environment is marked by several social interactions and requires academic competence, adaptability, and resilience to deal with challenges. During this phase, young individuals are susceptible to various implications that can impact their mental health and even cause disorders due to excessive study load, social, academic, and/or self-imposed pressure, difficulty in public speaking and/or socializing, and the need to live with unknown people and away from your family members [10,11].

Morever, it is important to describe the scenario in which the study was carried out. COVID-19 was an outbreak that originated in Wuhan, China, in 2019 and reached Brazil in February 2020. As of January 2022, within the ongoing data collection phase of this study, approximately 20 million individuals worldwide had been infected, and the death toll approached 76 thousand. In Brazil, confirmed cases totaled 1.2 million, with deaths reaching 4.6 thousand [41]. Among various restrictive measures, social isolation emerged prominently, potentially impacting mental well-being and emotional eating [28], and has led to heightened rates of anxiety and depression worldwide [42].

The high frequency of these disorders among Brazilian university students may be attributed to the substantial impact of the disease within the country. Brazil ranked among the top five nations in infection rates and held the second position globally in terms of deaths [41]. Moreover, at the time of the research, the pandemic had persisted for over a year, and university students were still experiencing remote education [25].

Emergency remote education may have also impacted students’ mental health, leading to decreased academic performance, higher failure rates, and an overload of academic tasks. Other challenges experienced by students include a lack of physical contact with colleagues, reliance on internet access quality, limited proficiency in specific tools, and concerns about transitioning back to face-to-face activities, as well as personal challenges like family illness or loss [17], and worries regarding family outcomes [43]. The onset of the pandemic beginning was characterized by fear and uncertainty, but, as the pandemic prolonged, anxiety about its end intensified [38,44].

Several studies have investigated the relationship between emotional eating and specific mental health conditions within young populations. For instance, Cecchetto et al. (2021) [28] investigated associations between emotional eating and the mental impact caused by social isolation in 365 Italians and found that increased emotional eating was predicted by higher depression and anxiety, while the increase in bingeing was predicted by higher stress. Liboredo et al. (2021) [29] analyzed associations between eating behavior through the TFEQ and perceived stress in 1368 Brazilians and identified that increased food intake and perceived stress were independently associated with emotional eating. Furthermore, Kalkan Uğurlu et al. (2021) [25] evaluated the relationship between mental disorders and eating behavior in nursing students (*n* = 411) and found that students enrolled in advanced periods have higher emotional eating scores, indicating that the longer time between the student’s bond with the university constitutes a stressor and has an impact on eating behavior.

The studies involving the university population [15,45] are limited to a singular course area or a few courses of study. In contrast, our present study adopts a more comprehensive approach, encompassing all undergraduate courses across the eight Federal Institutions of Higher Education involved in the research. It also includes participation from various course areas and college students at different stages of their academic journey. Thus, comparisons are limited by the differences in sample characteristics, researched outcomes, and the instruments used for evaluation.

It is acknowledged that, alongside mental disorders, university life leads to changes in eating habits and behaviors, with a common decrease in the consumption of fresh and minimally processed foods, an increase in the intake of alcoholic beverages, and changes in eating behavior, such as dysfunctional emotional eating [14,29]. Moreover, low-quality nutrition significantly impacts health and serves as a strong predictor of mental disorders [46].

Certainly, stress often influences unhealthy food preferences, and convenience may be a contributing factor. Individuals experiencing stress in their daily routines may opt for time-saving measures, avoiding the preparation of meals [14]. In stressful situations and people with depressive symptoms, it is common to replace one of the main meals with snacks [46], as well as the consumption of UPF, sweets, and treats [26,47] as comfort foods [48]. Additionally, the “serotonin hypothesis” suggests that the palatability of certain foods momentarily enhances well-being, alleviating negative emotions. However, this consumption does not necessarily indicate psychiatric problems. It is worth noting that the release of glucocorticoid hormones under stress may lead to seeking pleasurable or even compulsive activities, redirecting food choices to energy-dense options, preferably with a sweet taste, capable of modulating serotonin levels [3,14].

Moreover, a poor-quality diet could be a cause or a consequence of mental and eating disorders because of inadequate nutrient intake. Some nutrients such as complex B vitamins (B6, B9, and B12), amino acids (tryptophan, phenylalanine, tyrosine, histidine, choline, and glutamine), and marine-derived omega-3 fatty acids are essential for neurotransmitter production [49]. The preference for unhealthy foods may reflect a low emotional regulation [50,51].

Is it important to emphasize that, in this study, the relationship between emotional eating and mental health symptoms may be bidirectional. Psychological distress can lead to increased emotional eating as a way to manage emotions, while dysfunctional eating patterns may, in turn, exacerbate anxiety and depressive symptoms [6,14,28]. Previous studies suggest that the frequent consumption of UPF may negatively impact mood, reinforcing this vicious cycle [47,48]. Future longitudinal studies are essential to better understand these associations and develop targeted intervention strategies.

Furthermore, another factor that acts as a trigger to disinhibit food self-control in adverse situations or stressful events is the intentional practice of dietary restrictions, and this disinhibition can occur as a coping strategy [1,27,28,29]. Considering the food beliefs explained by the cognitive–behavioral model, it is possible that these stressful events, such as those experienced during academic life, in interpersonal relationships, or the pandemic period, caused changes in behavioral responses with the unconscious intention of seeking emotional balance [6,51].

Although this study investigated the associations between emotional eating and symptoms of anxiety, depression, and stress in Brazilian university students, it is essential that we consider how cultural factors may influence this relationship and the generalization of results to other populations. Studies conducted in different countries suggest that emotional eating patterns can vary due to cultural, social, and environmental factors that affect stress coping mechanisms and eating habits [9,52]. For example, research in Europe and the United States links emotional eating with the consumption of UPF high in sugars and fats as a response to negative emotions [9,35].

Additionally, the relationship between emotional eating and mental health can be influenced by socioeconomic factors. In Europe, financial and academic issues are associated with a higher propensity for emotional eating [53]. This is consistent with our findings, as socioeconomic vulnerability is also an important factor for Brazilian university students. However, countries with better student support policies and access to healthy eating may experience less of an impact from these factors on eating behaviors.

Research in Espana shows trends similar to Brazil, suggesting that the link between mental health symptoms and emotional eating may be common among young adults [54]. Thus, although the findings of this study align with international research on emotional eating and mental health symptoms, cultural and socioeconomic differences must be considered when applying these results to other populations. Multicenter and cross-cultural studies would provide a deeper understanding of the factors influencing emotional eating in various contexts.

Furthermore, personal and social factors, including gender [50], age [55], family income [53,56], body composition [1,57], area of study [15,25], physical activity practice [15], and UPF consumption [47], also impact the relationship between mental health and eating behavior by triggering negative feelings and emotions. In this study, these variables were used as adjustment factors, reinforcing the connection between emotional eating and mental health as observed in the multiple regression analyses.

Possible changes in the eating behavior of college students, including dysfunctional levels linked to the symptoms of mental disorders, are worrisome given the short-, medium-, and long-term damage to the health of young people [26,58]. To minimize the incidence and occurrence of mental disorders and improve students’ quality of life, it is important that we value the mental health support centers available in academic spaces, including those in the universities involved in this study.

The current study has some limitations that should be acknowledged. This is a cross-section study, which limits our ability to establish causal relationships. In this context, the observed relationships may be bidirectional. The sample selection that relied on convenience increased the chances that only students interested in the subject would participate. Additionally, there was a loss of samples due to incomplete questionnaire responses, and the use of a virtual questionnaire might have induced memory bias, especially concerning questions referencing the past. In an effort to increase the response rate, reminder emails were frequently sent to eligible students, including those who had abandoned the questionnaire before completing it. In college surveys, a response rate between 5% and 10% is expected [59]. Our response rate is within the expected range and higher than that of other surveys conducted during the pandemic period [60].

In this study, BMI was estimated using self-reported data. Since women (who predominated in this sample) and individuals with higher BMI often tend to underestimate their body weight [61], this may have influenced the results, as it was used as an adjustment variable in this study. However, this approach is a common practice in various surveys in Brazil, such as the Surveillance System for Risk and Protective Factors for Chronic Diseases by Telephone Survey (Vigitel) and online surveys [27,28].

Despite some limitations, this study offers valuable insights into the relationship between emotional eating and symptoms of anxiety, depression, and stress among university students. The research is based on a robust and representative sample, encompassing students from all undergraduate courses across multiple universities of Minas Gerais. We specifically analyzed emotional eating in relation to the severity of anxiety, depression, and stress symptoms as independent variables linked to high emotional eating scores, an area that has been underexplored in prior research efforts.

Given these findings, future studies should consider adopting longitudinal designs to explore the causal relationship between mental health disorders and emotional eating behaviors. Incorporating objective measures of dietary intake, quality of life, and measured anthropometric measurements would further enhance the accuracy of the results. Furthermore, future investigations could delve deeper into the role of socioeconomic factors, and cultural influences. Finally, intervention studies that implement or evaluate the effectiveness of mental health and nutritional support programs in addressing eating behavior issues, especially those related to emotions, could provide critical evidence for designing targeted strategies to promote student well-being.

## 5. Conclusions

Through this study, significant associations between emotional eating and the signs and symptoms of mental disorders among university students were observed. Emotional eating scores increased with the severity of mental health symptoms, highlighting the complex interplay between psychological well-being and eating behaviors. These findings emphasize the interdependence of mental health and dietary habits, underscoring the importance of addressing them collectively to prevent potential misinterpretations and reinforcing the need for an integrated strategy that considers both aspects when promoting student health.

Eating behavior and mental health are multidimensional concepts that warrant further exploration in the scientific domain, especially considering the potential implications brought about by the COVID-19 pandemic. Seeking psychosocial support to manage the symptoms of mental disorders is crucial in preventing their exacerbation, which could significantly impact the future quality of life and health of these young individuals, potentially fostering “dysfunctional emotional eating”.

These results highlight the importance of implementing targeted interventions within university settings. Institutions should prioritize accessible mental health programs, nutritional counseling, and stress management strategies to help students develop healthier coping mechanisms. Additionally, integrating mental health and nutrition education into academic curricula or incorporating them into lectures and interactive activities could foster greater awareness and early intervention. Public health initiatives and policymakers should also consider the broader implications of these findings, as emotional eating linked to psychological distress may contribute to long-term health risks, including obesity and metabolic disorders.

## Figures and Tables

**Table 1:** Personal data and emotional eating of students enrolled in face-to-face undergraduate classes in the Federal Institutions of Higher Education of Minas Gerais–Brazil, in 2021 and 2022.

Personal Data (Categorical Variables)	Total Students (*n* = 8650) % (N)	Emotional Eating Median (IQ)
**Sex**		
Female	65.7 (5660)	50.0 (27.8–72.2) *
Male	34.3 (2955)	33.3 (11.1–61.1)
**Body Mass Index (classification)**		
Underweight	8.9 (770)	27.8 (5.6–44.4) ^a^
Normal weight	55.1 (4768)	38.9 (16.7–61.1) ^b^
Overweight	35.3 (3055)	61.1 (33.3–88.9) ^c^
**Course area**		
Health	23.8 (2061)	44.4 (27.8–66.7) *
Other areas	76.2 (6589)	44.4 (22.2–66.7)
**Physical activity**		
No	36.0 (3113)	44.4 (22.2–72.2) *
Yes	64.0 (5537)	44.4 (22.2–66.7)
**Consumption of UPF**		
No	3.1 (265)	33.3 (5.6–66.7)
Yes	96.9 (8385)	44.4 (22.2–66.7) *
**Personal data** **(numerical variables)**	**Total students (*n* = 8650)** **Median (IQ)**	**Emotional Eating** **(correlation)**
**Age (years)**	22.0 (20.0–25.0)	r = 0.033 *
**Family income (US$)**	733.33 (314.29–1571.43)	r = −0.057 *
**Body Mass Index (kg/m^2^)**	23.3 (20.5–26.7)	r = 0.349 *

UPF: ultra-processed food consumption; IQ = interquartile ranges; ^a–c^ Different letters indicate statistically different values (*p* < 0.01; Dunn’s post hoc test). Mann-Whitney, Kruskal-Wallis, and Spearman correlation tests were applied. * *p* < 0.01. Total students: sex *n* = 8615; BMI *n* = 8583; family income *n* = 8090.

**Table 2:** Absolute and relative frequencies of signs and symptoms of mental disorders among students enrolled in face-to-face undergraduate classes at Federal Institutions of Higher Education of Minas Gerais–Brazil, in total population and in students with emotional eating scores above the third quartile in 2021 and 2022.

Signs and Symptoms of Mental Disorders		Total Students (*n* = 8650) % (N)	Emotional Eating ≥ 66.7 (*n* = 2593) [% (N)]
**Anxiety**			
No (normal)		33.9 (2933)	20.0 (519) ^a^
Yes	Mild	6.4 (553)	5.4 (139) ^a^
	Moderate	16.2 (1405)	17.2 (445) ^a^
	Severe	9.5 (824)	10.1 (263) ^a^
	Extremely severe	33.9 (2935)	47.3 (1227) ^b^
**Depression**			
No (normal)		26.8 (2320)	14.3 (372) ^a^
Yes	Mild	10.2 (881)	8.6 (224) ^a^
	Moderate	18.2 (1574)	18.3 (475) ^a^
	Severe	12.3 (1062)	13.5 (350) ^b^
	Extremely severe	32.5 (2813)	45.2 (1172) ^c^
**Stress**			
No (normal)		31.9 (2761)	17.3 (449) ^a^
Yes	Mild	10.6 (918)	8.5 (220) ^a^
	Moderate	17.0 (1467)	17.3 (448) ^a^
	Severe	20.8 (1799)	26.1 (667) ^b^
	Extremely severe	19.7 (1705)	30.8 (799) ^c^

^a–c^ Different letters indicate different values (*p* < 0.01). Chi-square with pairwise analysis post hoc test using Bonferroni correction.

**Table 3:** Factors independently associated with scores above the third quartile of emotional eating of students enrolled in face-to-face undergraduate classes at Federal Institutions of Higher Education of Minas Gerais in 2021 and 2022.

	Odds Ratio (OR)	Confidence Intervals of 95% (CI)	*p*-Value
Emotional Eating ≥ 66.7 crude model (*n* = 8650)			
**Anxiety symptoms**	2.648	2.374–2.953	0.000
**Depression symptoms**	2.830	2.505–3.199	0.000
**Stress symptoms**	2.948	2.630–3.304	0.000
Emotional Eating ≥ 66.7 adjusted model (*n* = 8558) (73.1% of prediction; Hosmer–Lemeshow test = 0.155)			
**Anxiety symptoms**	1.488	1.291–1.714	0.000
**Depression symptoms**	1.707	1.469–1.983	0.000
**Stress symptoms**	1.725	1.483–2.006	0.000

Adjusted for sex, age, family income, physical activity, ultra-processed food consumption, body mass index classification, and course area in the multiple logistic regression.

## Data Availability

The datasets presented in this article are not readily available for ethical reasons. Requests for access to datasets should be directed to livia.ferreira@ufla.br.

## Abbreviations

The following abbreviations are used in this manuscript:

B12	cobalamin
B6	pyridoxine
B9	folic acid
BMI	estimated body mass index
CI	Confidence Intervals
COVID-19	Coronavirus Disease 2019
DASS	Depression Anxiety and Stress Scale
e.g.,	for example
et al.	and others
HREC	Human Research Ethics Committee
i.e.,	that is
IQ	interquartile ranges
kg/m^2^	kilograms per square meter
N or *n*	number of participants
OR	Odds Ratio
*p*	significance level
PADu	Project on Anxiety and Depression in University Students
Q3	third quartile
SPSS	Statistical Package for Social Sciences
TFEQ	Three-Factor Eating Questionnaire
UFJF	Federal University of Juiz de Fora
UFLA	Federal University of Lavras
UFMG	Federal University of Minas Gerais
UFOP	Federal University of Ouro Preto
UFSJ	Federal University of São João Del-Rei
UFU	Federal University of Uberlândia
UFVJM	Federal University of Vales do Jequitinhonha and Mucuri
UNIFAL	Federal University of Alfenas
UPF	Ultra-processed food
US$	United States Dollar
Vigitel	Surveillance System for Risk and Protective Factors for Chronic Diseases by Telephone Survey
WHO	World Health Organization

## Appendix A

**Table A1:** Emotional eating score and the classifications of signs and symptoms of anxiety, depression, and stress of students enrolled in face-to-face undergraduate classes at Federal Institutions of Higher Education of Minas Gerais in 2021 and 2022.

Signs and Symptoms of Mental Disorders	Emotional Eating Median (IQ)
**Anxiety**	
Normal	33.3 (11.1–55.6) ^a^
Mild	44.4 (27.8–66.7) ^b^
Moderate	44.4 (27.8–66.7) ^b^
Severe	47.2 (33.3–66.7) ^b^
Extremely severe	55.6 (33.3–83.3) ^c^
**Depression**	
Normal	33.3 (11.1–50.0) ^a^
Mild	44.4 (22.2–66.7) ^a^
Moderate	44.4 (27.8–66.7) ^b^
Severe	44.4 (27.8–72.2) ^b^
Extremely severe	55.6 (27.8–83.3) ^c^
**Stress**	
Normal	33.3 (11.1–50.0) ^a^
Mild	44.4 (22.2–61.1) ^b^
Moderate	44.4 (27.8–66.7) ^c^
Severe	50.0 (33.3–77.8) ^d^
Extremely severe	61.1 (33.3–94.4) ^e^

IQ = interquartile ranges; different lowercase letters represent significantly different values.
